# Perceptual Processing is Not Spared During the Attentional Blink

**DOI:** 10.5334/joc.20

**Published:** 2018-03-19

**Authors:** Alon Zivony, Shira Shanny, Dominique Lamy

**Affiliations:** 1 Tel Aviv University, IL

**Keywords:** Attention, Semantics, Word processing, Visual perception, Consciousness, Working memory

## Abstract

Identification of the second of two targets is impaired when these appear within 500 ms of each other. This phenomenon, known as the attentional blink (AB) is thought to reflect disrupted post-perceptual processing. Yet, decisive empirical support for this claim is lacking. We measured the depth of the AB, while manipulating the second target’s reporting feature. We reasoned that if perceptual processing is unaffected by the blink, all the features of the blinked target should have equal access to working memory. In contrast with this prediction, we found identity and semantic-category reports to be more severely impaired by the blink than color reports, although baseline performance was similar in the two report conditions. These findings suggest that high-level features are more poorly represented in working memory than low-level features during the blink. We conclude that the attentional blink disrupts perceptual processing. Implications for contemporary models of the attentional blink are discussed.

## Introduction

Our ability to extract important information from a rapidly changing environment is severely limited. For instance, when two events are presented in close succession, observers often fail to identify the second event. In the lab, this phenomenon, known as the Attentional Blink (AB, Raymond, Shapiro & Arnell, 1992), is typically demonstrated when two targets (T1 and T2) are embedded in a rapid serial visual presentation (henceforth, RSVP) stream and presented at a rate of approximately 100 ms per item. The deficit in identifying T2 follows a clear temporal pattern: it is often absent when T2 immediately follows T1 (T1–T2 lag 1), it is largest when T2 appears 200–300 ms after T1 (T1–T2 lags 2 and 3), and it completely dissipates after 600 ms (T1–T2 lag 6 and above).

There is currently no consensus as to what mechanisms underlie the AB. It has been suggested that it reflects structural limitations on the number of items that can be consolidated in working memory (e.g., Chun & Potter, 1995; Jolicœur & Dell’Acqua, 1998), withheld or delayed attentional engagement (e.g., Nieuwenstein, Chun, van der Lubbe & Hooge, 2005; Olivers & Meeter, 2008; Wyble, Bowman & Nieuwenstein, 2009), or disrupted attentional control (e.g., Di Lollo, Kawahara, Ghorashi & Enns, 2005; Taatgen, Juvina, Schipper, Borst & Martens, 2009). Despite such controversy, all contemporary models share the premise that the AB occurs at a late stage of processing, after all features of the second target, including its semantic properties, have been extracted (see Dux & Marois, 2009, for a recent review). In other words, these models advocate a *post-perceptual* locus of the AB.^1^

An early version of this claim can be traced back to the seminal paper by Chun and Potter (1995: 122), who suggested that during the rapid presentation of successive stimuli, “the categorical identity of most of the items, and probably their specific identity (see Sperling et al., 1971), is briefly available and may serve as the basis of selection into subsequent stages”. While subsequent theoretical accounts rejected other aspects of Chun and Potter’s model, they uncritically integrated the assumption that perceptual analysis is unaffected by the AB. Accordingly, the AB is thought to occur later and to reflect failure to transfer or consolidate incoming information in working memory (WM).

### Evidence in favor of the post-perceptual account

The first studies put forward as supporting this post-perceptual account showed that a blinked stimulus primed a subsequent semantically related target (Maki, Frigen & Paulson, 1997; Shapiro, Driver, Ward & Sorensen, 1997). However, these studies did not include a comparison between the semantic priming effects elicited by blinked vs. non-blinked stimuli. Thus, while they indicate that some high-level processing survives the blink, they do not demonstrate that perceptual processing is unimpaired by the blink.

Vogel, Luck and Shapiro (1998) provided the main piece of evidence demonstrating that perceptual processing is unaffected by the blink. These authors examined the N400, an event-related potential (ERP) component thought to reflect post-semantic processes (Kutas & Federmeier, 2011), when observers reported either T2 only (single task) or both T1 and T2 (dual-task) at varying T1–T2 lags. Vogel et al. (1998) found the N400 associated with T2 to be smaller in the dual than in the single task, but to the same extent within and outside the blink period. They concluded that “the lack of a decrement in N400 amplitude during the attentional blink in Experiment 2 can be taken as strong evidence for a post-perceptual effect of attention.” (Vogel et al., 1998, p. 1666).

Consistent with this conclusion, several behavioral studies reported that semantic priming is not impaired during the blink (Harris, Benito & Dux, 2010; Harris & Little, 2010; see also: Visser, Merikle & Di Lollo, 2005). For instance, Harris and Little (2010) had participants report the identity of two objects embedded in a stream of distractor objects. They found T2 accuracy to be higher following a semantically related vs. unrelated distractor, and equally so when the distractor appeared inside (Lag 2) and outside (Lag 6) the blink. Additional, albeit less direct support for the post-perceptual account comes from a study by Asplund, Fougnie, Zughni, Martin, and Marois (2014), who showed that the blink affected the likelihood of consciously perceiving the second target, but not the precision of its representation.

### Challenges to the post-perceptual account

Other studies challenge the notion that semantic processing is intact during the blink. First, attempts to replicate Vogel et al.’s (1998) consistently revealed that N400 is either reduced (Rolke et al., 2001) or abolished by the AB (Batterink, Karns, Yamada & Neville, 2010; Peressotti, Pesciarelli, Mulatti & Dell’Acqua, 2012; Pesciarelli et al., 2007). For example, using the same design as Vogel et al. (1998), Batternik et al. (2010) reported that the N400 was reduced during vs. outside the blink. In addition, they showed that the residual effect during the blink was significant when correct and incorrect T2 trials were entered in the analysis, yet was no longer reliable when only incorrect T2 trials were considered. Giesbrecht, Sy and Elliott (2007) suggested a possible resolution of these discrepancies. They manipulated T1 task difficulty and found that the N400 was intact when the T1 task was easy and reduced when the task was difficult (see Sy, Elliott & Giesbrecht, 2013, for similar results).

Likewise, in many behavioral studies, semantic priming either was reduced (Martens, Wolters & van Raamsdonk, 2002; Zivony & Lamy, 2016) or disappeared altogether (Murphy & Bloom, 2015; Rolke et al., 2001; Peressotti et al., 2012) during the blink. For instance, Peressotti et al. (2012) used the three-target paradigm developed by Shapiro et al. (1997), in which three targets are presented in one stream. While T1 is unrelated to the other targets, T2 can be either semantically related or unrelated to T3. The authors found that when T2 was accurately reported, T3 accuracy was higher following a related vs. an unrelated T2. However, no such effect was found when T2 was missed (i.e., when it was blinked). Elliot, Baird and Giesbrecht (2016) did not study semantic priming, but their results also that challenge the post-perceptual account. Participants reported both the color and identity of T2 and performance accuracy on one dimension was independent of accuracy on the other dimension. This finding suggests that the AB disrupts feature binding or a process that precedes it, which is suggestive of a perceptual locus of the AB.

However, it is important to point out that finding reduced semantic priming or N400 amplitude during the blink do not necessarily argue against the post-perceptual account. Instead, these findings might reflect disruption of processes that occur downstream from high-level perceptual processing. Indeed, semantic priming and N400 effects do not index only the extraction of semantic information, but also the efficiency of later processes, such as response selection mechanisms. Therefore, reduction of these semantic effects can be expected even if the AB only impairs post-perceptual processes. In other words, while finding semantic effects to be unaffected by the blink constitutes convincing evidence that the blink affects post-perceptual processes, finding reduced semantic effects does not necessarily entail that high-level perceptual processing is impaired during the blink.

### A novel approach

To summarize the current state of the literature, investigations of the effect of the AB on semantic processes have yielded an inconsistent picture. On the one hand, several studies show that processing of high-level features is not impaired during the blink (e.g., Harris et al., 2010; Harris & Little, 2010; Vogel et al., 1998; Visser, Merikle & Di Lollo, 2005). However, these findings may be restricted to weak instances of the AB, in which the T1 task is easy (Giesbrecht et al., 2007; Sy et al., 2013). On the other hand, the empirical strategy used to refute the idea that the locus of the AB is post-perceptual, is also open to alternative accounts: it relies on the modulation of the N400 and semantic priming, which may also reflect modulation of post-perceptual processing. Here, we suggest a novel approach to test whether the AB disrupts perceptual processing.

Studies investigating the locus of the AB focused on semantic processing because semantic processing presumably reflects the latest stage of perceptual processing. These studies rely on the well-established notion that during perceptual processing, low-level features such as colors and orientations are extracted first, and identification of high-level features such as complex shapes and semantic meaning occurs later (e.g., Itti & Koch, 2001; Treisman, 2014; Wolfe, 2014). Here, we relied on the same idea, yet instead of examining whether AB disrupts semantic processing, we investigated whether it affects high-level perceptual processing to same extent as it does lower-level perceptual processing. Low-level features are resolved very quickly (Li, 2002) and are perceived pre-attentively (Wolfe, 2014). Therefore, low-level perceptual representations are less likely to suffer from the blink. Accordingly, many electrophysiological studies showed that the AB does not modulate the sensory P1 and N1 components of the ERP, which index low-level feature detection (Koivisto & Revonsuo, 2008; Kranczioch et al., 2007; Lasaponara et al., 2015; Sergent, Baillet & Dehaene, 2005; Vogel et al., 1998).

Here, we reasoned that if the AB disrupts perceptual processing, this disruption should be more devastating for late than for early perceptual processes. Conversely, if the AB only disrupts post-perceptual processes, such disruption, by definition, occurs after lower-level and higher-level perceptual representations have been formed, at which point it no longer matters which perceptual feature (low-level or high-level) is resolved faster.

According to this rationale, the disrupted perceptual processing account and post-perceptual account yield differential predictions regarding the depth of the AB for different visual features. Here, we compared the effects of the AB on reporting a high-level feature (identity) vs. a low-level feature (color) of the same target (T2). According to the disrupted perceptual processing account, the AB should be deeper for identity reports than for color reports, because color processing occurs earlier than identity processing. In contrast, according to the post-perceptual account, T2 performance should be equally impaired by the blink, irrespective of whether the task is to report T2’s color or its identity, because the AB affects only post-perceptual processing of T2.

In Experiment 1, two targets (T1 and T2) were embedded in a rapid serial visual presentation (RSVP) stream, with either two or six intervening distractors between them (lag 3 or 7, respectively). T1 was a Landolt C and participants had to localize its open side. T2 was the only boldfaced stimulus in the stream. The main manipulation was what feature of T2 had to be reported: its color (report-color condition) or its identity (report-identity condition). The depth of the blink was measured as the decrement in T2 accuracy at lag 3 relative to lag 7. These lags were selected because disruption of T2 processing is thought to peak at lag 3 (300 ms) and to subside entirely at lag 7 (700 ms), which can thus serve as a baseline (e.g., Harris & Little, 2010). With this measure, we could examine whether the level of processing required for the reporting feature (color or identity) affects the depth of the blink (as predicted by the disrupted perceptual processing account), or does not (as predicted by the post-perceptual account).

## Experiment 1

### Method

#### Sample size selection

Based on a pilot experiment,^2^ we calculated the sample size required in order to observe a significant interaction between task (color vs. identity) and T1–T2 lag (3 vs. 7). We conducted this analysis with G*Power (Faul, Erdfelder, Buchner & Lang, 2013), using an alpha of 0.05, power of 0.80, and the effect size found in the pilot experiment (η^2^_p_ = 0.30). We found the minimum sample size required to be 8 (i.e., 4 per group). Nevertheless, we used 26 participants, which yielded 99% power.

#### Participants

Participants were 26 (18 women) Tel-Aviv University undergraduate students who participated for course credit. The participants’ mean age was 23.26 (SD = 1.16). All reported normal or corrected-to-normal visual acuity and color vision.

#### Apparatus

Displays were presented in a dimly lit room on a 23” LED screen, using 1920 × 1280 resolution graphics mode and 120Hz refresh rate. Responses were collected via the computer keyboard and mouse. Viewing distance was set at 50 cm from the monitor.

#### Stimuli and design

**Figure 1:**
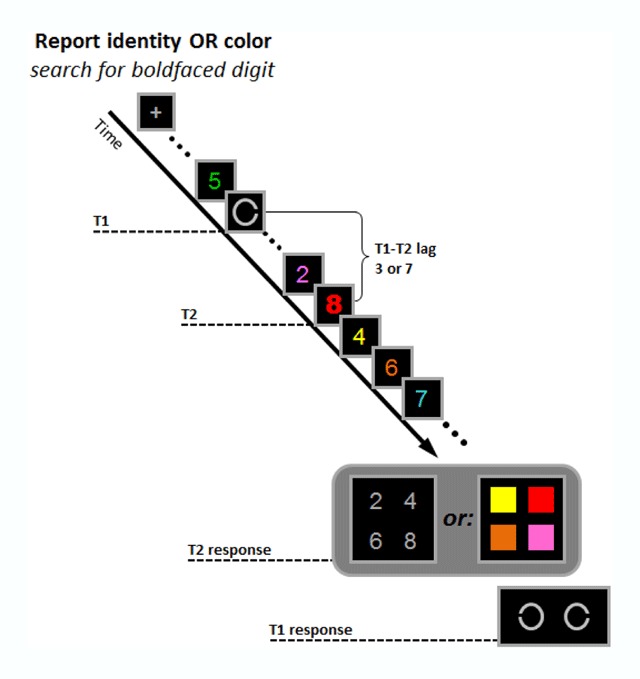
Illustration of the stimulus sequence in Experiment 1. The first target (T1) was a Landholt C and subjects had to determine whether it was open to the left or right. The second target (T2) was the only boldfaced stimulus in the stream. The response feature of T2 (identity or color) was manipulated between subjects.

The sequence of events on each trial is presented in Figure 1. The fixation display was a gray 0.2° × 0.2° plus sign against a black background. The stimulus sequence consisted of an RSVP stream of 18 frames. One frame contained the first target (T1), a grey circle subtending 1.2° in diameter and with a 0.2° gap (Landolt C) on either its right or its left side. Each of the remaining frames contained a colored digit. The digits were 2, 3, 4, 5, 6, 7, 8 or 9 and their color was red, pink, yellow, orange, purple, blue, cyan or green. The second target (T2) was written in the boldfaced “Arial black” font and subtended 1.1° in height and approximately 0.6° in width. The remaining digits (the distractors) were written in “Arial” font and subtended 1° in height and approximately 0.5° in width. The identities of T2 and of the three surrounding distractors (T–1, T+1, and T+2) were drawn randomly without replacement from four possible digits (2, 4, 6, 8) and their colors were drawn randomly without replacement from four possible colors (red, pink, orange, yellow).^3^ These four digits and four colors appeared only once per stream. The remaining distractors were selected randomly with replacement from the four remaining digits (3, 5, 7, 9) and colors (blue, purple, cyan, green), with the restriction that no color or digit could repeat in two subsequent frames.

The response display for T2 consisted of the four possible target digits (2, 4, 6, 8) drawn in grey for the report-identity group and of the four possible target colors (red, pink, orange, yellow) presented as 1° × 1° filled squares for the report-color group. For both groups the stimuli in the response screen were arranged in square configuration, 4.4° in side. The response display for T1 consisted of the two possible T1 stimuli, appearing 2° to the left and right of fixation.

Half of the participants were allocated to the report-identity task group and the other half to the report-color task group. The experiment included 10 practice trials followed by 480 experimental trials divided into 60-trial blocks. Subjects were allowed a self-paced rest between blocks.

#### Procedure

Each trial began with a 500ms fixation display followed by a 500ms blank screen and then by the RSVP stream. Each frame appeared for 41.66ms and was followed by a 58.33 ms blank screen. T2 appeared randomly at the 10^th^, 12^th^ or 14^th^ temporal position. T1 appeared either three or seven frames prior to T2. Participants had to identify whether the gap in the T1 Landolt C was on its right or left side. Participants searched for T2, defined by its boldfaced font. In the report-identity group, they reported its identity, whereas in the report-color group, they reported its color. Participants first responded to T2 by clicking their answer on the response display with the mouse. The response display for T1 appeared immediately after the first response. Participants responded to T1 by pressing the “x” key if the gap was on the right and “z” if it was on the left, on a standard keyboard. Participants were asked to guess if unable to identify the targets. None of the responses were speeded. Eye movements were not monitored but participants were instructed to focus their gaze on the fixation point. A new trial began 500ms after response.

### Results

One participant was removed from the sample because her accuracy both outside and inside the blink was lower than chance (M = 23.4% and M = 24.6%, respectively). Follow-up debriefing revealed that she did not understand the instructions. In this and the following experiments, all results remained similar when an arcsine-square root transformation was applied to the accuracy data.

#### T2 accuracy

**Figure 2:**
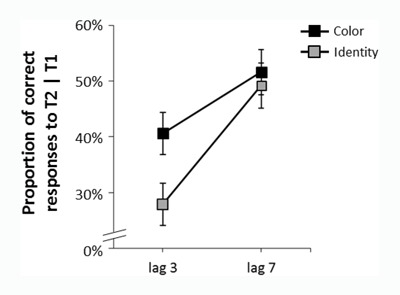
Average T2 accuracy rates, for correct T1 responses, as a function of task and T1–T2 lag in Experiment 1. The AB was deeper in the report-identity than in the report-color task. Error bars denote between-subject standard errors.

Mean T2 accuracy rates are presented in Figure 2. We conducted a two-way Analysis of Variance (ANOVA) with task (report identity vs. report color) as a between-subjects factor, T1–T2 lag (3 vs. 7) as a within-subject factor and T2 accuracy as the dependent variable. All T1-error trials were excluded from this analysis. The main effect of T1–T2 lag was significant, F(1,23) = 58.33, p < .001, η^2^_p_ = .72, and the main effect of task was not, F(1,23) = 1.71, p = .201, η^2^_p_ = .06. Importantly, the interaction between the two factors was significant, F(1,23) = 5.17, p = .032, η^2^_p_ = .18. Follow-up analysis revealed that the effect of T1–T2 lag was significant for both tasks, but was larger in the report-identity task, M = 27.8% vs. M = 49.2%, for lag 3 vs. lag 7, respectively, F(1,23) = 47.23, p < .001, η^2^_p_ = .68 than in the report-color task, M = 39.3% vs. M = 50.9%, for lag 3 vs. lag 7, respectively, F(1,23) = 14.99, p < .001, η^2^_p_ = .39. Thus, the AB was deeper in the report-identity task than in the report-color task. We also conducted a planned comparison to compare accuracy at baseline (lag 7) in the report-color and report-identity tasks, and found no difference between them, F < 1, η^2^_p_ = .0004. For the sake of completeness, we also analyzed the effect of task at lag 3 and found accuracy to be significantly lower in the report-identity than in the report-color task, F(1,23) = 4.63, p = .042, η^2^_p_ = .16.

#### T1 accuracy

The same two-way ANOVA with T1 accuracy as the dependent measure yielded no significant main effect, F < 1 and F(1,23) = 2.26, p = .14, η^2^_p_ = .09, for task and T1–T2 lag respectively, and no interaction between the two factors, F < 1. Mean T1 accuracy rates are presented in Table 1.

**Table 1:** Mean percentage of correct responses to T1 in Experiments 1–2, as a function of task and T1–T2 Lag (standard errors are presented in parentheses). Standard errors are between subjects in Experiment 1 and within subject (Morey, 2008) in Experiment 2.

	Report-color		Report-identity	

	Lag 3	Lag 7	Lag 3	Lag 7

Experiment 1	91.0 (2.3)	89.6 (2.6)	91.9 (1.7)	90.8 (2.3)
	**Report-color**		**Report-category**	

	**Lag 3**	**Lag 7**	**Lag 3**	**Lag 7**

Experiment 2	93.3 (0.7)	93.0 (0.9)	90.9 (1.0)	91.1 (1.0)

### Discussion

The results of Experiment 1 show that the attentional blink disrupted T2 identity reports to a larger extent than T2 color reports. This finding is incompatible with the claim that perceptual processing is spared during the blink. Moreover, performance was similar and below ceiling in the two report conditions at lag 7 (i.e., at baseline). This finding is crucial because it suggests that overall task difficulty was similar in the two tasks. Thus, differences in T2 task difficulty cannot account for the difference in AB depth observed between the two report conditions (e.g., Elliot et al., 2016). These findings support the disrupted perceptual processing account of AB.

## Experiment 2

The objective of Experiment 2 was to generalize the findings of Experiment 1 in two respects. First, in Experiment 1, the distractors that surrounded the target (T–1, T+1, T+2) always had the possible target colors and identities (see footnote 3), which may have alerted participants to the target’s presence. In Experiment 2 we removed any constraints on the distribution of response-relevant distractors in the RSVP stream. Second, we compared the report-color task to a task that requires more extensive processing than the identification of overlearned stimuli such as digits, namely, to a word categorization task (animals, plants and objects).

### Method

#### Sample size selection

Based on the results of Experiment 1 we calculated the sample size required in order to observe a significant interaction between task (report color vs. report category) and T1–T2 lag (3 vs. 7). We conducted this analysis with G*Power (Faul et al., 2013), using an alpha of 0.05, power of 0.80, and the effect size found in the previous experiment (η^2^_p_ = 0.18). We found the minimum sample size required to be 12 overall. Note that this analysis relied on an interaction between a within- and a between-subjects factor, whereas the design in Experiment 2 was fully within-subject and therefore had more statistical power. Nevertheless, we included 24 participants, which yielded 99% power.

#### Participants

Participants were 24 (16 women) Tel-Aviv University undergraduate students who participated for course credit. The participants’ mean age was 23.00 (SD = 2.65). All reported normal or corrected-to-normal visual acuity and color vision.

#### Apparatus, stimuli and design

**Figure 3:**
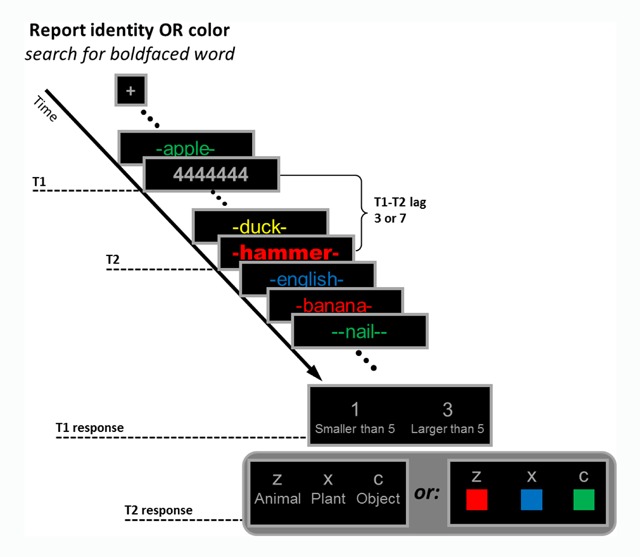
Schematic illustration of the sequence of events in Experiment 2. The first target (T1) was a gray string of eight repetitions of the same digit, and subjects had to determine whether this digit was smaller or larger than 5. The second target (T2) was the only boldfaced stimulus in the stream. The response feature of T2 (category or color) was manipulated within subjects. The actual stimuli were written in Hebrew.

**Figure 4:**
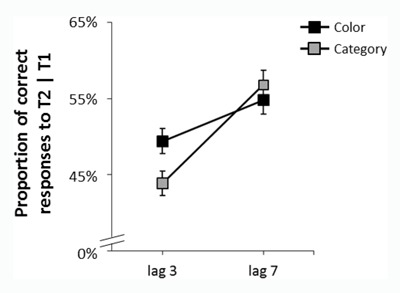
Results of Experiment 2. Average T2 accuracy rates for correct T1 responses, as a function of task and T1–T2 lag. The AB was larger in the report-category than in the report-color task. Error bars denote within-subject standard errors (Morey, 2008).

The apparatus, stimuli and design were similar to those of Experiment 1 except for the following changes. The stimuli set included 80 Hebrew words drawn from four possible categories: animals, plants, objects and abstract words (e.g., “lion”, “banana”, “hammer”, and “law”, respectively). All words had a frequency of 2 per million and above (based on a corpus of Israeli blog posts, Linzen, 2009) and were 3–6 characters long. Word stimuli were flanked by two “-” characters if they were 3- or 4-character long and by one “-” character if they were 5- or 6-character long. All stimuli other than T2 were written in “Arial” font, and subtended 1° in height and 3.5° to 6° in width. The first target (T1) was a gray string of eight repetitions of the same character, randomly drawn from eight possible digits (1, 2, 3, 4, 6, 7, 8, 9). The second target (T2) was written in the boldfaced “Arial black” font, subtended 1.2° in height and 4° to 6.5° in width, and was selected randomly from one of three possible categories (animals, plants, or objects). The color of T2 was randomly selected from three possible colors (red, green or blue). The remaining distractors were drawn with repetition from the four possible lists (animals, plants, objects, or abstract words) and the four possible colors (red, green, blue or yellow) with the restrictions that a specific word appeared only once per stream and the same semantic category or the same color could not occur in two consecutive frames. The response screens for T1 and T2 included the possible responses corresponding to the task (see below) arranged in horizontal configuration around fixation (see Figures 3 & 4). The exposure duration of each frame and the inter-stimulus interval (ISI) was 50 ms, which kept overall frame rate the same as in Experiment 1.

#### Procedure

The procedure was similar to that of Experiment 1 except for the following changes. First, the task was manipulated as a blocked variable within subjects, and task order was counterbalanced between subjects. For each task, 40 practice trials were followed by 100 experimental trials divided into two 50-trial blocks. Participants were allowed a self-paced rest between blocks. They were required to take a longer break after the second experimental block, during which instructions for the second task were provided. Second, the T1 task was to identify whether the digit in the T1 frame was smaller or larger than 5, by pressing the “1” key if it was smaller than 5, and the “3” key if it was larger than 5, with their right hands. Third, the T2 task was to identify the semantic category of T2, while disregarding its color in the report-category condition, and to identify T2 color, while disregarding its identity in the report-color condition. Participants responded to T2 with their left hands using the “z”, “x”, and “c” to indicate “animal”, “plant” and “object” in the report-category condition, or “red”, “blue” and “green” in the report-color condition. Finally, the response screen for T1 appeared immediately after the RSVP stream and was followed by the response screen for T2.

### Results

Two participants were removed from the sample because debriefing revealed that they actively tried to read T2 in the report-color task despite instructions not to. They were replaced by two new participants. Preliminary analyses revealed no effect involving task order, word length or word frequency, all Fs < 1. Therefore, the results were collapsed across these conditions.

#### T2 accuracy

We conducted a two-way ANOVA with task (report category vs. report color) and T1–T2 lag (3 vs. 7) as within-subject factors, and T2 accuracy as the dependent variable. The main effect of T1–T2 lag was significant, F(1,23) = 48.30, p < .001, η^2^_p_ = .68, and the main effect of task was not, F < 1. The interaction between the two factors was significant, F(1,23) = 11.30, p = .003, η^2^_p_ = .33. Follow-up analysis revealed that the effect of T1–T2 lag was significant for both tasks, but was larger in the report-category task, M = 43.9% vs. M = 56.9%, for lag 3 vs. lag 7, respectively, F(1,23) = 61.34, p < .001, η^2^_p_ = .71, than in the report-color task, M = 49.4% vs. M = 54.8%, for lag 3 vs. lag 7, respectively, F(1,23) = 9.94, p = .006, η^2^_p_ = .28. Similar to Experiment 1, there was no difference in accuracy between the report-color and report-category tasks at lag 7, F < 1, η^2^_p_ = .01. For the sake of completeness, we examined the effect of task at lag 3 and found it to approach significance, F(1,23) = 3.63, p = .069, η^2^_p_ = .15.

#### T1 accuracy

The same two-way ANOVA with T1 instead of T2 accuracy as the dependent measure yielded no significant main effect of task, F(1,15) = 2.30, p = .14, η^2^_p_ = .09, T1–T2 lag, F < 1, or interaction between these factors, F < 1 (see Table 1).

The AB was deeper for the report-category than for the report-color task. We thus extended the main finding of Experiment 1 to a semantic categorization task and to a within-subject manipulation of task category with no constraints on the type of distractors temporally flanking T2.

## General Discussion

We reexamined the widely embraced claim that the attentional blink does not impair perceptual processing. Previous studies that challenged the post-perceptual account of the AB relied on the finding that semantic priming and the amplitude of the N400 component are reduced during the blink. Our motivation for conducting the present study stemmed from the observation that such reduction can occur even if fully processed semantic information is available for post-perceptual processing. Therefore, these results can be explained by the post-perceptual account of the AB and do not provide a decisive test for it.

Here, we adopted a different approach and measured the cost of selecting a first target on performance at reporting a low-level vs. a high-level feature of a subsequent target. According to the post-perceptual account, the AB disrupts processing after all features have been resolved, and therefore should have the same impact on the report of low-level and high-level features. On the other hand, if the AB disrupts perceptual processing, such disruption should be more damaging for perceptual processing of high-level features than for perceptual processing of low-level features. Our results supported the latter account: the blink impaired identity as well as category reports more severely than color reports. We conclude that during the blink, perceptual processing is weakened, such that high-level features are more poorly represented than low-level features.

### Alternative accounts

The interplay between T2 salience and the strength of the post-T2 mask is known to strongly modulate the blink depth (e.g., Chua, 2005; Giesbrecht et al., 2003; Giesbrecht, & Di Lollo, 1998). One could therefore argue that the deeper blink observed here for identity than for color reports might result from T2 identity being less salient and easier to mask than its color. If it were the case, however, lower accuracy for identity and category relative to color reports should be observed irrespective of T1–T2 lag, because neither T2 salience nor the post-T2 mask varied as a function of lag (see also Chua, 2005, Experiment 3). Yet, in neither experiment was accuracy lower for identity or category than for color at lag 7, and in both, accuracy was clearly below ceiling at lag 7. Therefore, differences related to salience or masking cannot account for our findings.^4^

The relationship between the T1 and T2 tasks is also known to modulate the blink depth. Specifically, the blink is deeper when T1 and T2 involve different tasks than when they involve the same task (e.g., Chun & Potter, 2001; Di Lollo et al., 2005; Kawahara, Zuvic, Enns & Di Lollo, 2003). Therefore, differential similarity between the T1 and T2 tasks in the report-color and report-identity conditions might account for our findings. Note however that in these studies, the critical switch occurred from one defining feature for T1 to a different defining feature for T2. Here, in contrast, the defining feature of T1 was the same in all conditions and so was the defining feature of T2 in both experiments. Thus, the switch between the T1 and T2 tasks was the same for the report-color and report-identity conditions. Yet, even if a switch between the response features of T1 and T2 also deepened the blink, it would actually entail a shallower blink in the report-identity than in the report-color conditions. Indeed, if anything, the shape identification required in order to respond to T1 (orientation of a Landolt C in Experiment 1 and digit categorization in Experiment 2) was more similar to the digit identification and word categorization tasks required in the report-identity and report-category conditions, respectively, than to the color discrimination required in the report-color condition.

Finally, our perceptual account of the AB posits that once the representation of an object is formed, there is no difference in the post-perceptual processing of low- and high-level features. One could argue that encoding high-level features of a stimulus in working memory (i.e., post-perceptual processing) requires more attentional amplification than encoding its lower-level features. According to this account, high-level features might be more vulnerable to the blink, even if perceptual processing is unaffected by the blink. This suggestion entails that individual features of the same object enter WM separately and that feature binding therefore occurs after WM encoding. However, this idea is at odds with the findings of several recent studies showing that bound objects are encoded in WM (e.g., Luria & Vogel, 2011, see also Allen, Hitch, Mate & Baddeley, 2012; Karlsen, Allen, Baddeley & Hitch, 2010; Morey & Bieler, 2013). In addition, since semantic processing typically requires feature binding (e.g., here, between the features that made up a letter and between the letters that made up a word), the notion that unbound features enter WM would imply that semantic information becomes available only after encoding in WM – a conclusion that clearly contradicts a basic tenet of the post-perceptual account of the AB.

A post-perceptual framework might nevertheless be compatible with our findings if it posits that the AB unbinds an object’s color and identity but does not affect the binding between features that make up a letter or word and provides the basis for semantic processing. Although such an account is less parsimonious than the disrupted perceptual processing account advocated here, it may be useful to test it in further research.

### Relation to other models

The finding that perceptual processing is impaired during the blink is at odds with the notion that the blink reflects only the limited capacity of working memory (Chun & Potter, 1995; Jolicœur & Dell’Acqua, 1998). However, other models suggest that the blink results from disruption of attentional processes.

Some authors suggested that control over the attentional set is lost during the blink (e.g., Di Lollo et al., 2005; Taatgen, et al., 2009), such that early attentional processes cannot be initiated by T2. However, several studies reported that attentional control is intact during the blink (e.g., Nieuwenstein, 2006; Zivony & Lamy, 2014, 2016). For instance, we showed that an object matching the target’s defining feature captures spatial attention to the same extent whether it appears within or outside the blink (Zivony & Lamy, 2016).

Other authors proposed that the blink either suppresses or delays attentional engagement (e.g., Nieuwenstein et al., 2005; Olivers & Meeter, 2008; Wyble et al., 2009) after an attentional episode has been triggered. These theories emphasize the role of attentional engagement in working memory consolidation, yet, they can be easily modified to accommodate our finding that perceptual processing is disrupted during the blink, if they incorporate the notion that attentional engagement also enhances perceptual processing (see also Chua, Juliana & Nicholas, 2001). For example, consider the computational episodic simultaneous type serial token (eSTST) model proposed by Wyble and colleagues (Bowman & Wyble, 2007; Wyble et al., 2009). This model suggests that visual representations that include all feature information (*types*) are rapidly extracted based on bottom-up saliency and their match with the attentional set. In order to bind types and temporal information (*tokens*) into stable representations and store them in working memory, attentional engagement is required. In the eSTST model, types are represented as nodes with all features having equal status. For the model to incorporate our findings would only require the added tenets that (1) attentional engagement enhances the efficiency of perceptual processing; and (2) the type activation threshold for any given feature depends on how demanding the perceptual processing of this feature is, with the consequence that lower-level types are more likely to be activated and tokenized than higher-level types.

By positing that attentional engagement during the blink is weakened, this modified eSTST model provides a simple account for the results of the present study as well as resolves apparent disparities in the AB literature. Firstly, by stipulating that attentional engagement is not entirely suppressed during the blink, the model predicts that some semantic processing should occur during the blink. This explains why semantic priming effects are not completely eliminated during the AB, but rather shrink to various degrees in different studies (Batterink et al., 2010; Giesbrecht et al., 2007; Martens et al., 2002; Rolke et al., 2001; Zivony & Lamy, 2016). By stipulating that attention is engaged after the detection of T2, but peaks after it’s disappearance (Nieuwenstein et al., 2005; Olivers & Meeter, 2008; Wyble et al., 2009), the model predicts that the post-T2 distractor will be clearly represented, instead of T2 (Bourassa, Vachon & Brisson, 2015). Therefore, when T2 is embedded among distractors that have the possible reporting features, most T2 errors result from intrusions of the post-T2 distractor, that is, of instances where participants report the post-T2 distractor instead of T2 (Chun, 1997; Vul, Nieuwenstein & Kanwisher, 2008). Accordingly, in line of the disrupted perceptual processing account, the AB might affect the precision of T2 representation but this effect is masked by post-T2 intrusions (thus providing an alternative interpretation of Asplund et al.’s (2014) findings). Finally, it is important to note that on top of enhancing perceptual processing, attentional engagement is also likely to facilitate working memory consolidation (e.g., Vogel et al., 2005), which explains why the attentional blink is also reported for low-level features (Elliott et al., 2016; Jolicœur, Sessa, Dell’Acqua & Robitaille, 2006; Ross & Jolicœur, 1999). Accordingly, the few studies that reported intact semantic processing (Harris et al., 2010; Harris & Little, 2010; Vogel et al., 1998) during the blink might reflect instances where: (a) the low task demands from T1 allowed attentional engagement to T2, to a degree that was sufficient for perceptual processing but not post-perceptual processing (Giesbrecht et al., 2007); or (b) visual features were simple enough to be rapidly resolved despite impaired attentional engagement. Intact semantic priming from distractor object pictures (Harris et al., 2010; Harris & Little, 2010) might reflect the latter option, as object pictures can often be rapidly categorized based on their low-level features (Rogers & Patterson, 2007).

## Conclusion

In this study, we found that low-level features are more likely to survive the attentional blink than high-level features. These results challenge the widely held assumption that the attentional blink is entirely post-perceptual, and suggests instead that perceptual processing is disrupted during the blink.

## Data Accessibility Statement

Data from all experiments is available at figshare (https://doi.org/10.6084/m9.figshare.5492611).

## Additional File

The Additional file for this article can be found as follows:

10.5334/joc.20.s1 Supplementary materials. Distractor Intrusion Analysis.

## Footnotes

Following Vogel, Woodman and Luck (2005; see also: Treisman, 2014; Itti & Koch, 2001), we define perceptual processing as the operations involved in forming sensory representations. In vision, these operations include extracting basic visual features, binding them into more complex representations, and matching these to previously stored information. Thus, according to this definition, post-perceptual processing occurs after extraction of semantic information and includes encoding in working memory.

This pilot experiment was similar to Experiment 1 with the main difference being that in the report-color condition, the defining feature of the target was identity and its response feature was color, whereas in the report-identity condition, the defining feature was color and the response feature was identity. This manipulation generated the interaction predicted by the disrupted perceptual account, yet baseline performance (at lag 7) differed in the two report conditions, presumably because the defining feature was different. This observation led us to use the same defining feature in the two conditions, in Experiments 1 and 2.

This manipulation allowed us to examine the probability of erroneously reporting the distractors surrounding the target in time instead of the target (see Supplementary materials).

One could nevertheless claim that masking might interact with the AB, such that it might impair identity processing more severely than color processing inside but not outside the blink. However, this claim is actually consistent with the disrupted perceptual account because it implies that the AB renders semantic information more vulnerable to perceptual overwriting.

## Author Information

Support was provided by the Israel Science Foundation Grant 1475/12 to Dominique Lamy.
