# Supplementary material for: Perceptual Processing is Not Spared During the Attentional Blink
Source: J Cogn. 2018 Mar 19;1(1):18. doi: 10.5334/joc.20 (PMC6634390; doi:10.5334/joc.20)
Supplement: Supplementary materials. — Distractor Intrusion Analysis. [file joc-1-1-20-s1.pdf]

Supplementary materials

Distractor Intrusion Analysis

Distractor intrusions refer to erroneous reports of a distractor instead of the target. They are thought to occur due to the misbinding of target-defining features and available response features (Botella, Barriopedro & Suero, 2001). Intrusions from the distractors that are temporally adjacent to the target provide an indirect measure of the availability of the response features under varying levels attentional enhancement. Specifically, while distractors that immediately follow the target benefit from the spill-over attentional enhancement that accrues to the target, distractors that precede the target do not (Olivers & Meeter, 2008; Wyble et al., 2009). Accordingly, previous research has shown that the attentional blink leads to a redistribution of distractor intrusions: during the blink, intrusions from distractors that precede T2 (pre-T2) become less frequent relative to intrusions from the distractor that follows the target (post-T2), presumably because fewer attentional resources are available at that time (e.g., Chun, 1997; Vul, Nieuwenstein & Kanwisher, 2008).

As a supplementary analysis, we monitored the likelihood of pre-T2 (T2-1) distractor intrusions relative to the two post-T2 distractors (T2+1 and T2+2). In Experiment 1, there were four possible responses and the response features of distractors T2-1, T2+1 and T2+2 were selected with replacement from the pool of possible response features. Crucially, these distractors were the only distractors that could take on the potential response features and were therefore the only possible sources of intrusions. We examined whether the likelihood of making a pre-T2 distractor intrusions was affected by T1-T2 lag and the task. We predicted T2-1 intrusions should be more frequent in the

report-color than in the report-identity task and to a larger extent during than outside the blink.

We conducted an ANOVA with task (report-identity vs. report-color) as a between-subjects factor, and T1-T2 lag (lag 3 vs. lag 7) as a within-subject factor, with the proportion of pre-T2 distractor intrusions out of all error trials (i.e., the percentage of error trials in which T2-1 was reported instead of T2) as the dependent variable.

Results

Pre-T2 distractor intrusions were more frequent in the report-color than in the report-identity task, $F(1,23) = 8.25$, $p = .009$, $\eta^2_p = .26$, and at lag 7 than at lag 3, $F(1,23) = 10.23$, $p = .004$, $\eta^2_p = .26$. The interaction between the two factors was not significant, $F < 1$. Mean identity intrusion data are presented in Table 1.

Table 1. Mean percentage of pre-T2 errors (out of all errors), as a function of task and T1-T2 Lag. Between-subjects standard errors are presented in parentheses.

	T1-T2 lag	
T2 task	Lag 3	Lag 7
Report-color	22.4 (2.4)	29.5 (2.6)
Report-identity	14.2 (2.5)	21.7 (2.7)

Discussion

The proportion of pre-T2 intrusions (out of all errors) was lower for the identity- than the report-color task. This result is consistent with the claim that color is processed faster than identity, and is therefore more likely than identity is to be available during the blink, that is, when attentional enhancement is reduced. In addition, as reported in earlier

studies (e.g., Chun, 1997; Vul et al., 2008), the proportion of pre-T2 distractor intrusion errors was smaller inside than outside the blink.

However, in contrast with our prediction, the shift in pre-T2 intrusions was of similar magnitude in the report-color and report-identity conditions. The following observations can explain this finding. The redistribution of distractor intrusions during the blink is thought to occur because the representations of items appearing before T2 are weaker than the representations of items following T2 (e.g., Chun, 1997; Wyble et al., 2009). Thus, the proportion of pre-T2 distractor intrusion errors reflects the representation strength of this distractor's response feature *relative to* those of post-T2 distractors' response features (Botella et al., 2001). In the present design, intrusion errors could only come from T2-1, T2+1 and T2+2. This explains why the representation of T2-1 color relative to the representations of T2+1 and T2+2 colors was impaired to the same extent as the representation of T2-1 identity relative to the representations of T2+1 and T2+2 identities, even if, in absolute terms, processing the identity of the pre-T2 distractor was more impaired during the blink than processing its color.

Supplementary references

Botella, J., Barriopedro, M., & Suero, M. (2001). A model of the formation of illusory conjunctions in the time domain. *Journal of Experimental Psychology: Human Perception and Performance*, *27*(6), 1452-1467.

Chun, M. M. (1997). Temporal binding errors are redistributed by the attentional blink. *Attention, Perception, & Psychophysics*, *59*(8), 1191-1199.

Olivers, C. N., & Meeter, M. (2008). A boost and bounce theory of temporal attention. *Psychological review*, *115*(4), 836-863.

Vul, E., Nieuwenstein, M., & Kanwisher, N. (2008). Temporal selection is suppressed, delayed, and diffused during the attentional blink. *Psychological Science*, *19*(1), 55-61.

Wyble, B., Bowman, H., & Nieuwenstein, M. (2009). The attentional blink provides episodic distinctiveness: sparing at a cost. *Journal of Experimental Psychology: Human Perception and Performance*, *35*(3), 787-807.
